# Perceived Health System Challenges of Implementing Cross-Border Malaria Preventive Measures at Ports of Entry in KwaZulu-Natal

**DOI:** 10.5334/aogh.3992

**Published:** 2023-04-27

**Authors:** Ida M. Munsense, Joyce M. Tsoka-Gwegweni

**Affiliations:** 1 Department of Public Health Medicine, School of Nursing & Public Health, College of Health Sciences, University of KwaZulu-Natal, South Africa; 2 Faculty of Health Sciences, University of the Free State, Bloemfontein, South Africa

**Keywords:** cross-border, malaria preventive measures, imported malaria, implementation challenges, South Africa, KwaZulu-Natal

## Abstract

**Background:**

Cross-border movements, especially from a malaria-endemic neighbour, contribute to importation of malaria, as they provide favourable conditions for malaria transmission in the receiving country. In the KwaZulu-Natal (KZN) province of South Africa (SA), the uMkhanyakude district is one of the endemic malaria areas where the borders are characterised by frequent cross-border movements of travellers coming into the province, mostly from Mozambique. Many studies have suggested that regional efforts through the implementation of cross-border measures are needed in both the high- and low-endemic countries to effectively address imported malaria. The implementation of cross-border measures to prevent imported malaria has led to a significant decline in malaria cases in KZN and SA; however, those measures are subjected to various challenges.

**Objective:**

This study sought to determine the health system challenges of implementing cross-border preventive measures for imported malaria at the Kosi Bay, Kwaphuza and Golela ports of entry in KZN.

**Methods:**

This inquiry consisted of a mixed methods approach, of which the qualitative component is reported here. In-depth interviews were conducted with four purposively selected health officers working at the legal and illegal ports of entry of the KZN province. Data were coded manually and then analysed using thematic data and descriptive analyses.

**Results:**

This study identified operational and prevention challenges. The related operational challenges included travellers’ non-disclosure and refusal, uncontrolled cross-border movements and poor coverage as well as shortage of staff. The prevention challenges included lack of novelty in the existing cross-border preventive measures, insecurity and illegal migration. Concerning travellers’ non-disclosure and refusal to cooperate, these issues occur at the legal ports of entry of Kosi Bay and Golela, where travellers were less cooperative in disclosing their health-related information to health border officers. They were more eager to cross and attend to their business. The findings revealed a lack of new ideas in the existing cross-border measures for the prevention of imported malaria, which some scientists considered as the reason for the failure of the elimination efforts in SA. Because of the porous borders and the shortage of staff to cover all the uncontrolled entries, travellers constantly crossed without any hindrances. Porous borders exposed the people living at the border areas and travellers to insecurity, promoted criminal activities and encouraged illegal migration.

**Conclusion:**

Cross-border malaria preventive measures are meant to contribute to decreased travel-related disease. Failure to attain this purpose must be carefully examined and mitigation strategies implemented. The study revealed the challenges of implementing cross-border measures at the KZN ports of entry of Kosi Bay, Kwaphuza and Golela. The challenges occurred at the operational and prevention levels, which, if not effectively addressed, could impede the decrease of imported malaria in the malaria-endemic district of KZN and SA in general.

## Introduction

Cross-border movements have been one of the main causes of malaria outbreaks in South Africa, which in general is a low-endemic country [1]. The risk of outbreaks is always present due to unusually high rainfall and cross-border movement of migrant populations, which contribute to local transmission [2]. According to the Department of Home Affairs (DHA), in January 2020, 3,930,440 travellers, South African residents and non-residents, crossed the SA ports of entry, with the majority of them travelling by road [3]. KwaZulu-Natal is one of the three malaria-endemic provinces in SA [4]; its borders are characterised by cross-border movements from the endemic neighbouring countries [5]. Cross-border movements, especially from the malaria-endemic neighbouring areas, provide favourable conditions for malaria transmission, increasing imported malaria in the host country [6]. In the face of unavoidable cross-border movements, previous studies have suggested that the fight against malaria should be considered a regional issue, which requires the involvement of the receiving low-endemic country and the high-endemic neighbours. Regional efforts through cross-border initiatives have led to the decrease of malaria in the participating countries [7,8]. Although these initiatives have contributed to the decrease of imported malaria [9], they have their own challenges.

In southern Africa, the Lubombo (Zimbabwe, Zambia, Eswatini, South Africa, Namibia, Mozambique, Botswana and Angola) Spatial Development Initiative (LSDI) was developed between Mozambique, SA and Swaziland (now known as Eswatini) [10]. Through implementing vector evidence-based control, malaria prevalence and case management contributed to the decrease of malaria; however, the major challenges have been a lack of political goodwill and financial constraints. The latter is concerned about countries’ overreliance on international donors. From the political perceptive, it was found that there is discordance between policy decisions made at the national level not being explained to the technical and operational participants [8]. At the China-Myanmar border and its Myanmar and Chinese units, a good relationship of trust and cooperation between participating countries was a key element in the success of the cross-border programmes [11].

Also, there is sometimes a lack of collaboration with regard to data sharing, reluctance to adopt new innovative technologies, delays in the procurement and deployment of essential commodities due to the misalignment between national budget cycles and the seasonality of malaria, in most cases leading to significant delays in emergency responsiveness [12]. The lack of coordinated regional efforts results in the continued importation of malaria into the elimination areas [7]. Besides vector control measures and case management, regional initiatives have also used malaria screening of travellers crossing the ports of entry to prevent imported malaria. Evidence shows that malaria screening programmes reduced the risk of indigenous transmission in countries such as Mauritius [13]. In Mpumalanga, screening and treatment were used to complement other malaria control measures and were found to contribute to a decrease in the imported malaria cases. However, a positive screen will need adequate testing tools, treatment and possibly referral of travellers. Since the treatment is likely to be a multiple-dose regimen, travellers receiving the treatment should be encouraged to complete the dosage [14]. Furthermore, malaria screening works well where border crossings are tightly controlled, but it may be ineffective when there are uncontrolled ports of entry where people travel unchecked between countries [9]. In general, border areas are remote, less developed and often likely to have weak health systems. The level of surveillance in the border areas is low and insufficient to cover mobile and migrant populations [15].

In KwaZulu-Natal (KZN), the cross-border initiative between Mozambique, SA and Eswatini (MOSASWA) has implemented strategies to address asymptomatic malaria importation in both source and sink areas [7]. This initiative aimed to reducing transmission in southern Mozambique by strengthening human capacity to effectively control malaria and improving intervention coverage in the Maputo, Gaza and Inhambane provinces. The second objective focused on detecting and treating malaria infections before they reached receptive areas within KZN [16]. However, poor cross-border collaboration, weak political goodwill, insufficient resources and equipment and inadequate staff capacity are major concerns [17].

Other studies suggest that the MOSASWA initiative needed to be strengthened because of the constant risk of importation from the high-endemic areas and countries [18]. There has been extensive literature on malaria in SA, with most focusing on malaria elimination [18,19,20,21,22]. In evaluating the progress, however, very little research focused on cross-border measures to prevent imported malaria, which is one of the requirements for achieving elimination. This study aimed to determine the health system challenges of implementing the cross-border measures to prevent imported malaria at the Kosi Bay, Kwaphuza and Golela ports of entry into KZN, SA to provide evidence-based solutions that could help improve these measures.

## Rationale

The significance of this inquiry is evident. Several studies have shown the importance of treating asymptomatic carriers of malaria and that this is justified as a means to relieve the burden of the disease [23], particularly in areas marked by higher prevalence and exposure to malaria. Preventive measures and prophylactic treatment of this disease are vital because it is deadly, as the World Health Organization (WHO) describes it, ‘a life-threatening disease caused by parasites that are transmitted to people through the bites of infected female *Anopheles* mosquitoes’ [24].However, a few countries in Africa have structures and facilities to control the health status of people crossing borders, from one country to another. This study aimed to establish the challenges to implementing cross-border malaria preventive measures at ports of entry to prevent malaria import into SA.

## Methods

This qualitative research only included service providers at the border entry points. This was to report on the health system challenges of implementing cross-border malaria preventive measures at ports of entry. The intention was to later report on the views of the travellers as service users in order to present views from both sides. The latter component is a survey, which is yet to be completed. A qualitative research design is concerned with establishing answers to the whys and hows of the phenomenon in question and provides insightful information. This is unlike a quantitative design, which seeks to obtain quantifiable or generalizable data [25]. Data collected from qualitative research cannot usually be analysed in a quantifiable way using statistical techniques because there may not be commonalities between the various collected findings [26].

This study explored the views of the government-employed health officers working at the ports of entry at Kosi Bay, Kwaphuza and Golela in the uMkhanyakude district of KZN, SA. The aim was to gain a deeper understanding of the health system challenges in the implementation of cross-border measures for the prevention of imported malaria in KZN.

A purposive sampling technique was employed. This is a non-probability sampling strategy in which participants are strategically chosen based on pre-selected criteria relevant to the research questions [27]. A maximum of four in-depth interviews were conducted using a general interview guide. The key informants comprised two environmental health practitioners, a chief environmental health officer and a chief health inspector, who were available and voluntarily agreed to participate in the study. All four participants worked within the Department of Health (Port Health) and were familiar with the phenomenon of imported malaria, cross-border movements and the border preventive measures.

The participants were briefed about the objectives of the study. Detailed study information was provided in the informed consent for the participants to make an informed decision on whether they should participate in the study or not. The participants read and signed the consent forms before the interview. The interviews were conducted in English at the participant’s workplace, in a setting that ensured privacy and confidentiality. Following consent, all the interviews were recorded on audio tape, and the duration was between 12 and 46 minutes. The researcher transcribed the interview verbatim; this enabled familiarisation with the data and reflection on the emerging themes. Different recurrent unifying concepts or statements about the health system challenges of implementing cross-border preventive measures were identified and organised to gain more insights apparent from the whole dataset [28]. The emerging identified themes and subthemes were manually coded.

## Ethical Considerations

Permission to conduct this study was obtained from the Ministry of Health in KZN (Ref: KZ- 201811-013), and ethical approval was obtained from the Biomedical Research Ethics Committee (BREC) of the University of KZN (BREC Ref No: BE382/18). All respondents’ information was kept confidential, and electronic data was password protected. Personal identifiers were not stored with the data to ensure compliance with the Protection of Personal Information Act (POPI Act).

## Results

The findings of the study on the perceived health system challenges of implementing the cross-border measures for the prevention of imported malaria in KZN were divided into two themes and six subthemes, as presented in Table 1.

**Table 1:** Challenges of cross-border malaria preventive measures at KZN ports of entry.


THEMES	SUBTHEMES	PORT OF ENTRY

Operational challenges	Travellers’ non-disclosure and refusal	Controlled

	Uncontrolled cross-border movement and poor coverage of preventive measures	Uncontrolled

	Shortage of staff	Uncontrolled

Prevention challenges	Lack of novelty in the preventive measures	Controlled

	Insecurity and illegal immigration	Uncontrolled


*Source*: Qualitative data collected at the ports of entry of Kosi Bay, Kwaphuza and Golela in September 2019.

### Operational challenges

#### Traveller’s non-disclosure and refusal

The participants shared their concerns about the lack of cooperation from the travellers crossing the port of entry, whose first priority was to get into SA regardless of their health conditions. Participant 1 commented as follows:

The challenges that we have in this office is whereby a person does not want to give you the information. Some other hide that they are sick, but you have to go deeper and find the symptoms that are related to the disease that you are looking for. Therefore, they do not want to talk about anything related to their health. The only concern is they want to go [to] SA, whether sick or not; they want to be there. (Participant 1)

Another participant perceived travellers crossing at Golela, the controlled border crossing, to be immune to malaria infection, suggesting that their resources enable them to take precautionary measures against mosquito bites. Due to a time constraint, malaria screening is not that important for them, and testing for malaria at the legal border is not compulsory. Participant 4 commented as follows:

People entering through legal borders have no time for testing. They have all the resources to prevent mosquito bites. They sleep in the best places, which provide for mosquito repellents. They enter borders in a hurry with their cars and no laws forces them to get tested. Most of the people are from other countries [and most] are educated. They know what they need to provide. They cooperate very easily. We have never had a problem. (Participant 4)

At the uncontrolled border crossing where malaria testing takes place, the presence of soldiers compels the travellers to abide by the rules and thus address the issue of refusal:

In the past people used to say, I will not test. I am OK. They used to refuse. Working closely with soldiers has helped a lot. If the person does not want to test, we will say go back to your country. So far, that is the best way because people are crossing illegally. (Participant 1)

The same participant shared her discontentment with the lack of collaboration between the rank manager and the health practitioners after seeking assistance from the taxi manager concerning testing the travellers at Manguzi taxi rank:

At Manguzi Ithala Centre, we spoke to the rank manager, asking if they can test maybe at the taxi rank. They were having a challenge because the rank wants money, maybe R1000 or R1500. I am not sure about the exact amount for us to test there because anyone who partakes in doing any activity at the rank need to pay. That is how best we can do. They said that we are worried as you are concerning about people, but for you to work, do anything, even testing, you must pay. We said we could not pay to offer services to the community. (Participant 3)

#### Uncontrolled cross-border movement and poor coverage of preventive measures

Travellers cross the SA border for various reasons, such as business, work and tourism. Seemingly, those entering illegally for business or work cross anytime and do not take precautions, while travellers such as tourists use the legal border crossings. The latter are perceived as more knowledgeable of travel-related diseases and can take the necessary precautions for risk avoidance.

People travel a lot. That is a challenge. We work from 8:00 a.m. to 4:00 p.m. Other people can cross earlier or later. People do not use this border only; there are other borders that they cross illegally. For instance, Kulubeni, Esheni, Mbagweni [and] Musa are illegal entries that they use. People love SA a lot. They say it is full of opportunities. They do their market around [the] Mannguzi area. They open their businesses. It is an opportunity for them to make money. (Participant 3)

A participant shared her concerns about the uncontrolled movements of travellers regardless of the presence of soldiers and police. She believed that poorly controlled borders could be a source of imported malaria and also of many other travel-related diseases:

They use the borderline to cross. Although there is the South African National Defence Force (SANDF) and the police, they still cross the borderline. That is the biggest loophole whereby people can go in and out (SA and Mozambique) without control, without seeing what kind of the disease they have. Even Ebola maybe will come through the borderline. Who knows. The only thing that they are doing they just upgrade the borderline fence, but they are more specifically [concerned] with the vehicle theft. The main concern for them to renew the fence is for the cars not to cross, although there is a lot of people that are crossing the fence. (Participant 2)

Another participant pointed out the negative impact of uncontrolled cross-border movements on malaria control efforts in KZN and the government’s reluctance to address the issue of illegal entries. The participant highlighted a shortage of resources (human and financial) to provide cross-border services daily and an inability to control the illegal entries:

There is a lot of malaria coming from Limbane and Xaixai. Illegal border [crossing]is a political thing because the government is aware of it. They are the one that is letting it happen, but it [is] causing us a lot of havoc in terms of malaria control efforts in KZN. Along here, there are four illegal border posts: Kwaphuza, Mozi, Mbagweni, and Nkonjani. The four illegal border posts are open 24/7. People can walk as they wish anytime. The police and soldiers are there, and people are crossing as they wish, anytime day and night. I do not know why government is allowing that. At those illegal borders, they do testing, but not every day. Like I said Kwaphuza (Wednesdays and Saturdays), Mbagweni (Tuesdays and Thursdays), Nkojani, there is none, and people just cross anytime. They just go there sometimes because they cannot be there always. They can be there daytime, but at night, they will not be there. So people can cross anytime. (Participant 2)

Another participant echoed the occurrence of uncontrolled cross-border movements, stating the involvement of the regional initiative in the malaria control efforts to target the hot spot areas:

They also got a team—something to do with SADC [Southern African Development Community] thing of malaria. They do have a team, but I am not sure where they are working at for now. Because last time I checked they were working at gate 6, where people do small scale trading on Sunday and Wednesday. There are many movements of people that are not being controlled, so they were stationed there by that time. Now I am not sure if that SADC program for South Africa and malaria is still there. This container is for something, same as the one that are doing South African side, but they are not stationed here at the port of entry because they know that there is one on the Mozambican side. So they target those places whereby they test on spot and treat on spot. For the area of uMmhlabuyalingana, there is the team. (Participant 1)

A participant pointed out the impact of the porous borders and uncontrolled cross-border movement from endemic areas of Mozambique on the SA efforts to eliminate malaria, suggesting that the control efforts in SA will only be successful if the same efforts are made in Mozambique as well:

In fact, we are mandated to eliminate malaria. But due to those porous border posts, we are having a challenge because we will not be able to eliminate malaria soon because we can try everything in our area, but most people are coming from Mozambique with the parasite and we do have vectors also in SA. The only thing that we do not have much is the parasite. Once a person from Mozambique come[s] with the parasite and brings malaria, we will not be able to reach elimination. Because with elimination, we need not to have a local case. Once you have a case, you are not eliminating malaria. Even if you eliminate, you have to sustain it for a period of three years without having one single case, which is not possible. We are trying very hard. (Participant 3)

**Figure 1:**
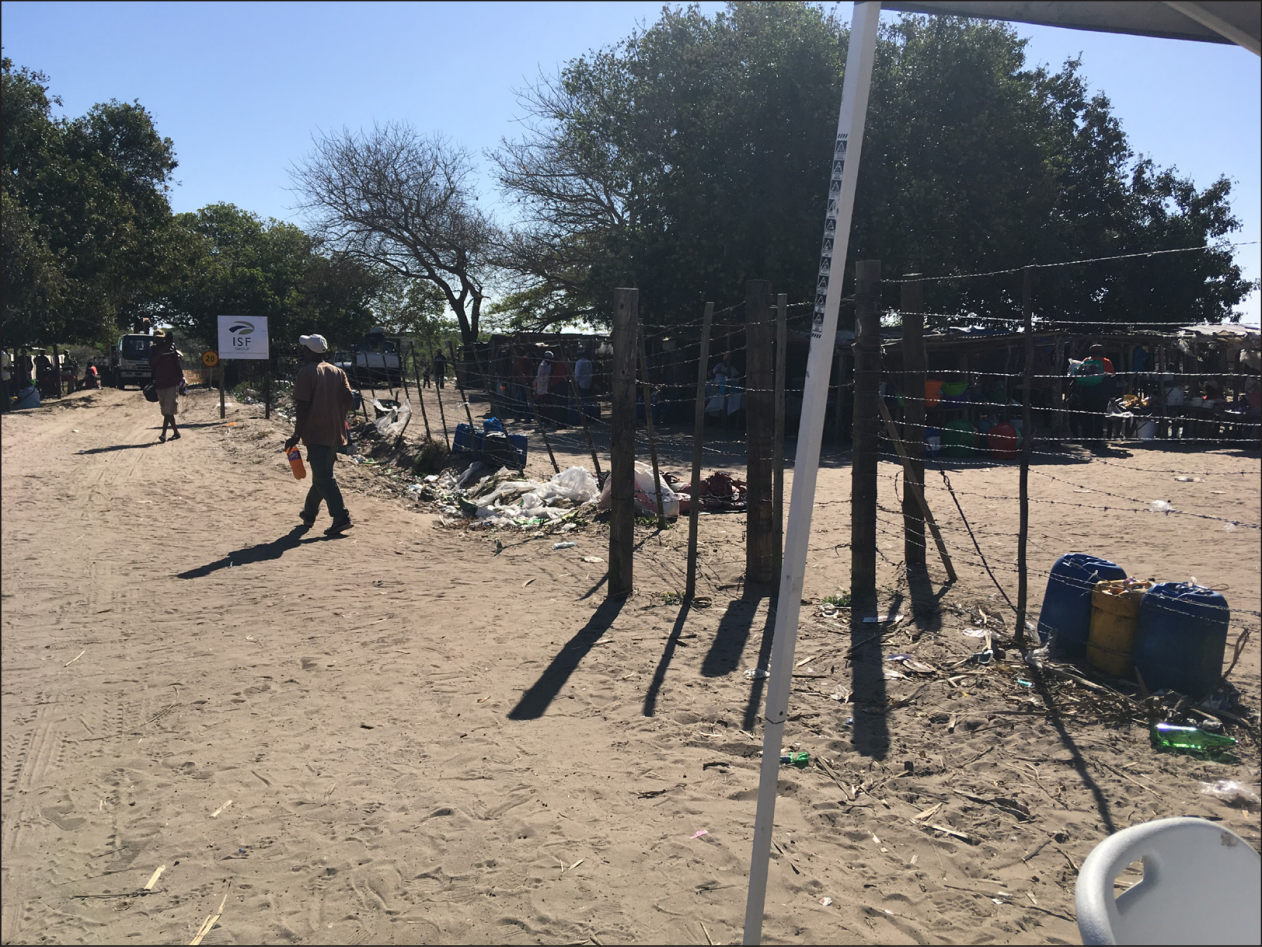
**Showing the condition of the Kwaphuza illegal port of entry where people cross without being checked or screened for malaria**. Porous border post at Kwaphuza port of entry. *Source*: Principal investigator’s camera picture taken at Kwaphuza port of entry 11/09/2019 at 14:26.

Participant 1 confirmed the weak infrastructure and the presence of several uncontrolled ports of entry besides the four known illegal border crossings as showed in Figure 1 below:

The fence is very loose. It is not a strong fence. It is very weak, and you can jump anywhere. So if the fence can be tougher that can solve the problem. The fence must not just allow people to jump across it. It must be difficult to cross. There are four known uncontrolled borders and so many other[s], maybe more than 100, where people just cross the fence. If you drive along the fence, there are big patches where people just move, cross the fence and go. The fence itself it is not good; it is weak. So if [the] government want[s] to protect us, the fence must be strong enough. That thing is political; everyone is aware. In Kwaphuza every Wednesday, we do not get no less than 15 positive malaria cases. (Participant 1)

#### Shortage of staff

Due to a staff shortage, the Department of Health collaborates with other partners, such as Humana People to People, to help with the screening at the border; however, these resources are still not enough to cover all the illegal entries. Participant 2 commented as follows:

We started about three to four years back. We have a team from Humana People to People. It is partners for the Health Department. The Department of Health did not have much staff. They asked [for more] from the World Health Organization (WHO) and national department. They got help from Humana People to People, so there are teams here in Jozini. There are only two teams. They screen people at the border, but they are not enough. (Participant 2)

### Prevention challenges

#### Lack of novelty in the existing cross-border preventive measures

There were concerns of the need for new approaches in dealing with imported malaria due to the lack of novelty in the existing cross-border preventive measures. This means the same implementation strategies that were used prior to 2018 are still being used, which is seen as a challenge to achieve malaria elimination in SA.

According to our strategic planning, we said we want to eliminate malaria in 2023. In fact we were targeting 2018, but we failed to eliminate in 2018; now we have moved to 2023. I do not see us winning because there nothing different that we are doing. For instance, if you want to do something new, you take different approaches. (Participant 2)

#### Insecurity and illegal migration

Participant 2 shared concerns about the safety of the population around a porous border and the inability of the government to address the issue of illegal entry:

Illegal borders do not come only with malaria; they come with many terrible things: drugs, cars theft and terrible other things. Anything you can think of which is bad comes from these illegal borders. So if they can close illegal borders posts, at least malaria can [be] reduce[d]. Everyone is aware of that. Even national is aware. WHO is aware of that. But they do not do anything. Our democracy is too much of a thing. It is dangerous. Our democracy is very too much. So once you come in South Africa you are at home; you will enjoy it. They are doing it deliberately. The government is failing us. (Participant 2)

Another participant added that porous borders encourage undocumented migrants. Illegal migrants avoid legal borders because of a lack of proper documentation; some go to the extent of settling down along the border areas, which puts a strain on health systems in SA by increasing imported malaria cases.

The office at Jozini is specialised with malaria. They are the ones that have the imported cases that they find in this area because some of the people do not have passport[s]; they do not use the border every time. There are many places along the borderline where they use to cross. Some of them stay near the border, where they build their houses to stay there, only to find that they increase the number of cases in the clinics [on] this side. If you trace a person, you will find out that he/she is not South African. Therefore, the challenge for now are the people, illegal immigrants, that are staying in South Africa without documents; [they] are the one[s] with the problem of malaria. (Participant 1)

A participant commented on the impact of illegal migration on imported malaria:

At the legal border of Kosi Bay, there [is] nothing. Most people come from Kwaphuza. Because it is an illegal border post, they do not use passport[s]. Very few people that come from far use Kosi Bay; most of them use Kwaphuza. People are coming further north of Mozambique, from Xai-Xai, Limbane. There they cross Kwaphuza. Those people are the ones with malaria. Out of 10 people coming from Limbane, maybe 8 are malaria positive. (Participant 2)

A participant added that illegal crossing is a practice well known to the soldiers, who allow the travellers to cross without proper documentation, but people like tourists use legal border crossings:

There are illegal borders, but I do not know the name because I heard some Swazi people are crossing, where they ask the soldiers and the soldiers allow them to pass without a passport. At this border of Golela, 90% of the travellers are tourists, but since the Kosi Bay side, their roads are open, it is working nicely, Mozambicans are going that side now. There are very few that are coming this side now. Mostly is tourist and Swazi people. (Participant 4)

Participant 3 added the following:

The government is failing us because they allow people to come illegally. If all these illegal border posts can be made legal and be able to employ people there day and night, if there are police there, you can send the staff to stay there for the all night. But without police and soldiers, we can’t risk people to stay there, so that’s our challenge. (Participant 3)

## Discussion

The study’s findings revealed the challenges associated with implementing effective cross-border malaria preventive measures at the Kosi Bay, Kwaphuza and Golela ports of entry. They also provide some thoughts for handling asymptomatic cases, which later may turn positive and infectious. This was not a serious issue at the time the research was conducted.

With regard to the cross-border preventive measures, the study found that travellers, especially those entering through the legal border crossings of Kosi Bay, were less cooperative when disclosing information concerning their health. They were more preoccupied with reaching their destination than making time for a health assessment with the health worker. At the legal border crossing of Golela, Participant 4 presumed that the travellers crossing the border are well off because of their resources. As most of them are tourists, they have a better knowledge of malaria and preventive measures; therefore, they do not need malaria screenings. These assumptions could be detrimental to the travellers’ health, because regardless of their knowledge, they could be asymptomatic [16]. Some studies found that travellers could carry infected mosquitoes in their cars or suitcases, which could cause an outbreak and contribute to onwards transmission in low-endemic areas [29].

Participants 1 and 4, working at the legal border crossing of Kosi Bay and Golela, respectively, said that malaria is not their only disease of concern. They provide health education on all travel-related diseases. Therefore, cross-border preventive measures, such as malaria screening, are non-existent at the legal border crossings of Kosi Bay (leading to Mozambique) and Golela (leading to Eswatini), and according to the participant 4, at the Golela port of entry, malaria screening is not compulsory.

Uncontrolled cross-border movement results from numerous illegal entries along the border where malaria screening and treatment services are not provided. This viewpoint agrees with a study in Yemen, bordering Saudi Arabia, which found that the large influx of an undetermined number of malaria-infected illegal migrants from Yemen might have led to the increase in locally acquired cases in the Jazan region of Saudi Arabia [6]. Porous borders have led to an uncontrolled cross-border influx of travellers from malaria-endemic countries, thus contributing to malaria transmission in low-endemic areas [9].

The findings also revealed a shortage of staff that can work 24/7 to monitor entries. This is a concern of not having enough environmental health practitioners to work at the illegal border crossing of Kwaphuza to screen travellers. Participant 2 revealed that the Department of Health sought help from partners to assist with screening activities at the illegal border crossings, but this was still not enough given the number of illegal border crossings. These findings are consistent with other research that found that the existing human resources were inadequate to effectively implement active surveillance activities in the three malaria-endemic provinces in SA [22].

There is a lack of novelty in the existing cross-border malaria preventive measures. According to participant 3, malaria control in KZN was still using the same strategies of fighting against imported malaria that were implemented in 2012, even though the province failed to reach the 2018 elimination target and reset for 2023. This is consistent with other studies, which found that the lack of new and innovative implementation strategies for effective malaria interventions were a cause of concern for malaria elimination efforts [30]. Another study found that the national malaria control programmes in SA are extremely wary of adopting and implementing new techniques and technologies [31].

The findings also revealed that the border was poorly controlled; there were many illegal ports of entry, which caused insecurity at the border areas and encouraged illegal migration in KZN. It was found that KZN was the most significant contributor to stock theft and ranked first for unsafe cities across SA [32]. People indulging in illegal activities often avoid official border posts [33]. A study conducted at Beitbridge found that undocumented Zimbabwean travellers bribed corrupt immigration officers and taxi drivers to cross the border to SA. However, once they crossed, they fell victim to human smuggling, which often resulted in robbery, sometimes in rape and death [34]. Robust, fences will contribute to the decrease of illegal activities and improve effective surveillance of the entries into SA.

This study revealed the lack of efficient cross-border measures to prevent imported malaria, which is considered by some scientists as the reason why efforts to eradicate malaria in SA are unsuccessful. Furthermore, inadequate fencing and the shortage of staff to cover all the uncontrolled entries where travellers constantly illegally find their way into SA without any hindrances are critical issues that call for attention for the following reasons:

- Firstly, this study sought to determine the health system challenges of implementing cross-border measures for malaria prevention in place at the Kosi Bay, Kwaphuza and Golela ports of entry. Besides pinpointing operational and prevention challenges, there was the necessity to look into cases of asymptomatic carriers among the population under investigation. It is important to acknowledge that ‘scaling up malaria control interventions has resulted in a substantial decline in global malaria morbidity and mortality’ [35].
- Secondly, incremental funding and commitment towards malaria prevention and treatment schemes across Africa has contributed to reducing cases of infections. However, it has become evident that more interventions are needed to satisfy the international target of reversing the growing malaria incidence [23]. These scholars have also advocated “the prospective role of an innovative malaria control strategy—the community-based treatment of asymptomatic carriers of *Plasmodium falciparum*—with Artemisinin-based combination therapy (ACT)” [23]. According to these scientists, the treatment of asymptomatic carriers with ACT is an innovative and pivotal tool for breaking the cycle of infection in some transmission settings [23]. They also argue that safe and efficient medication can save the lives, but that is only temporary, as mosquitoes can be reinfected from the asymptomatic carriers, because the presence of carriers is a threat to public health. Thus, the eradication of the parasite is close at hand because of improved and speedy diagnostic tests that ease the process of identifying asymptomatic carriers and eliminating the parasite [23].
- Finally, the lack of a comprehensive regional or continental strategy to prevent the transfer of malaria infection from one country to another was also remarkable. Thus, the revival of programmes such as LSDI and the MOSASWA malaria initiative is very much needed today because of the increase in infection incidence since the termination of LSDI in 2011 [36]. There is a growing need for the revival of MOSASWA-MI [36].

There should be policies for rapid testing of malaria to identify asymptomatic carriers and administer appropriate treatment to them. This strategy will significantly keep the malaria parasite out of SA’s borders. Other research has found similar evidence of asymptomatic malaria infection’s impact on malaria transmission and that interventions to target this parasite reservoir may be needed to achieve malaria elimination in both low- and high-transmission areas [35].

## Limitations

The present research showed a few limitations in terms of number of participants to collect empirical data and their availability. It is worth mentioning that several participants were invited to join the study but declined. It would have been prudent to include other sources, such as registries from the provincial malaria control office on imported malaria cases recorded in KZN on a monthly basis and nurses from the referral clinic to ensure that there is follow-up of the cases from the borders. The authors acknowledge the limitation of not presenting the views of travellers in this current study. However, a study is currently being completed and will provide the views of travellers as service users. Despite these limitations, the findings provided novel insights into the health system challenges of implementing cross-border malaria preventive measures in KZN, especially at the Kosi Bay, Kwaphuza and Golela ports of entry, which are the major borders in the province. The findings further provided valuable information on cross-border malaria preventive measures that could not be addressed with quantitative data.

## Conclusion and Recommendations

Implementation of effective cross-border preventive measures remains the surest way to control imported malaria originating from malaria-endemic neighbours. This study sought to determine the health system challenges of implementing cross-border measures for malaria prevention measures in place at the Kosi Bay, Kwaphuza and Golela ports of entry. The findings of the study revealed the operational and prevention challenges.

At the legal border crossing of Kosi Bay, there is a lack of cooperation from travellers concerning their health issues and a lack of novelty in the existing implementation strategies for cross-border measures for preventing imported malaria. Although travellers crossing at the legal border crossings were perceived to be immune from the infection due to their resources, they were less cooperative in disclosing their health information. Notwithstanding their resources, they could be asymptomatic and contribute to the onwards transmission and thus feed the reservoir in the low-endemic areas of SA. There are uncontrolled movements of travellers at the illegal border crossing due to several illegal ports of entry, and there is also a shortage of staff, specifically human resources for health, to perform control activities such as malaria screening. Porous borders endanger the safety of the people living near the border and promote illegal immigration into KZN.

It is envisaged that the Port Health authorities will learn from the implementation of the current national COVID-19 lockdown regulations and will enforce cooperation from travellers at the legal borders, including screening for malaria, thereby reducing imported infections. Although COVID-19 caused the disruption of malaria preventive measures [37], it demonstrated a global cooperation in faster data sharing and openness, with obvious benefits to public health and optimisation of public health interventions when the need is great [38]. This type of collaboration is needed as far as imported malaria is concerned. Thus, we make the following recommendations:

- Malaria control should increase screening at legal posts and provide ACT treatment to the diagnosed asymptomatic carriers of the disease.
- There should be a systematic programme aiming to target and eliminate the reservoirs of malaria infection in the proximity of the border post to prevent mosquitoes that get infected from asymptomatic travellers from spreading the parasite.
- It is imperative to develop new approaches to fight imported malaria and an effective collaboration with various stakeholders (private-public establishments); these include encouraging voluntary screening of malaria via effective and speedy diagnosis.
- Cooperation between SA and its neighbours should be encouraged to promote education on the deadly effects of the malaria parasite and to administer prophylactic treatment to travellers who show even mild symptoms of malaria infection.
- A border fence to control illegal movements across illegal borders should be reinforced to prevent the import of malaria in the country.
- More equipped and trained medical personnel should be placed at border posts to screen for malaria infection at both legal and illegal border crossings to track infectious cases and provide immediate anti-malarial treatment to them.
- Various illegal border crossings should be reduced to more manageable ones through better control and improving the border infrastructure will decrease illegal immigration and is essential to win the battle for malaria control and prevention.
- Future research should include designing and testing innovative strategies to implement effective cross-border interventions to prevent imported malaria. These should include better ways of delivering health education messages and malaria screening activities to increase coverage.
- It is urgent to develop and implement health protocols that focus on the small-scale cross-border traders, as they generally contribute to a good proportion of movements at the border crossings in the Southern African Development Community (SADC) region. Such border-crossing policies should be informed by the data on gender, age and other demographics of the small-scale traders and an understanding of the obstacles to cross-border commerce.
- To lead an effective campaign, cross-border collaborations and support from bodies such as the Common Market for Eastern and Southern Africa (COMESA) and SADC are highly recommended.
- Uncontrolled borders remain a big challenge in the SADC region, as with other regions in the continent. Regional bodies, including the African Union (AU), should be involved and play a crucial role by becoming critical of malaria prevention strategies that are failing and developing regional and continental interventions to mitigate porous borders that are a risk for the spread of infectious diseases.
- The WHO should intervene by scaling up funds to support malaria elimination schemes such as LSDI and MOSASWA. That could help curb malaria cases, which have increased since the termination of that programme in 2011; the need for reviving MOSASWA malaria initiative is rising. Such initiatives should be developed in COMESA, SADC and other regional bodies.
- Lastly, there is a need to strengthen health capacities at the land crossings utilising the learnings from the COVID-19 pandemic and possibly adapting best practices from Ebola management.

## Areas for Further Studies

In the southern African context, SA is perceived as economic hub that offers trading opportunities to its neighbours. Travellers from different occupations and professions, of which most include small-scale traders, come to buy goods from SA to resell back home and vice versa. Although an important point, our present study did not focus on the travellers’ occupations but how all the travellers who cross the border are affected or infected with malaria. Hence, their knowledge, attitudes and perceptions towards malaria and the preventive and cross-border measures are significant details that we highlighted in this report. Since malaria does not exclude anyone but rather infects everyone, future study could investigate cross-border traders to better understand the issues surrounding cross-border trading and its impact on malaria infection and prevention and asymptomatic malaria infection carriers.

A further investigation is recommended to identify the magnitude of asymptomatic carriers of the malaria parasite in the border areas. This is a key area of prevention—based on the existing literature that support medical treatment of travellers who have not developed the symptoms of malaria yet are carrying the parasite. This could be one reason, among others, for the South African population’s vulnerability to malaria and the spread of the parasite in the SADC, MOSASWA and COMESA countries, where malaria infection is endemic. The administration of prophylactic treatment and ACT could prove to be efficient in an anti-malaria campaign from cross-border travellers.

## Data Accessibility Statement

The datasets used and/or analysed during the current study are available from the corresponding author on reasonable request.

## Figures and Tables

**Table:** 

ACT	Artemisinin-based combination therapy
BREC	Biomedical Research Ethics Committee
COMESA	Common Market for Eastern and Southern Africa
DHA	Department of Home Affairs
KZN	KwaZulu-Natal
LSDI	Lubombo Spatial Development Initiative
MOSASWA	Mozambique, South Africa and Eswatini (Swaziland)
MOSASWA-MI	Mozambique, South Africa and Swaziland Malaria Initiative
SA	South Africa
SADC	Southern African Development Community
SANDF	South African National Defence Force
WHO	World Health Organization
